# Resources for screening the literature for glycan-related terms using PubAnnotation in GlyCosmos

**DOI:** 10.1093/glycob/cwag015

**Published:** 2026-03-09

**Authors:** Jin-Dong Kim, Masaaki Shiota, Issaku Yamada, Kiyoko F Aoki-Kinoshita

**Affiliations:** Database Center for Life Science (DBCLS), Research Organization of Information and Systems (ROIS), 178-4-4 Wakashiba, Kashiwa, Chiba 277-0871, Japan; Glycan and Life Systems Integration Center (GaLSIC), Soka University, 1-236 Tangi-machi, Hachioji, Tokyo 192-8577, Japan; The Noguchi Institute, 1-9-7 Kaga, Itabashi, Tokyo 173-0003, Japan; Glycan and Life Systems Integration Center (GaLSIC), Soka University, 1-236 Tangi-machi, Hachioji, Tokyo 192-8577, Japan

**Keywords:** annotation, bioinformatics, databases, glycans, literature mining

## Abstract

To facilitate access to relevant text of literature related to data in GlyCosmos, we have developed a collection of annotated literature resources using the agile annotation method supported by the PubAnnotation system. As a proof of concept, we compiled two dictionaries for glycan motifs and epitopes, plus six additional dictionaries for relevant biological entities, covering organisms, phenotypes, diseases, and anatomical locations. Next, we collected all the PubMed abstracts from 15 selected journals, and annotated them based on these eight dictionaries. This resulted in 279,368 annotation instances made to 15,463 abstracts, meaning that we were able to automatically pull glycan motif and epitope annotations related to diseases, taxonomy, etc. from over 15,000 abstracts. All the annotations were converted into Resource Description Framework (RDF) statements to support flexible querying. For users who are not familiar with RDF, we also developed a Web interface in GlyCosmos to visualize the location of the text in publications as well as query templates to personalize queries for specific terms. Pilot searches and analyses suggest that these resources are useful for navigation of relevant contexts of biomedical associations relevant to glycobiology.

## Introduction

The scientific literature is the foundation of scientific data. While scientific data curated in databases facilitate convenient searching and querying about certain biological data, further details about the data are often accessible only in the literature. As such, researchers often want to check the relevant literature for better understanding of scientific data and pieces of knowledge, and researchers of glycobiology are no exception. For glycobiologists, a literature search component has been added to the portal site of GlyCosmos (Yamada et al. 2020; Lee et al. 2025), using the PubAnnotation system (Kim et al. 2019). PubAnnotation provides an infrastructure for indexing the content of the literature based on a predefined list of terms appearing in it. Terms are defined in what are called Dictionaries, whereby specific terms are mapped to identifiers, such as NCBI Taxonomy IDs for organisms and GlyTouCan IDs for glycans.

The indexing is implemented in the form of annotations of the locations of specific text (keywords) in the literature, which is also known as “named entity annotation”. We have thus developed an infrastructure whereby glycan-related terms can be easily extracted from the literature using named entity annotation. This infrastructure includes components to (a) manage dictionaries of terms of interest (in PubDictionaries), (b) automatically annotate text based on the dictionaries (by PubDictionaries), (c) index the annotations in Resource Description Framework (RDF) format (in PubAnnotation), (d) access specific pieces of text with specific annotations (in PubAnnotation), and (e) visualize the annotations (using TextAE).

To enhance the accessibility of GlyCosmos-related literature, three specialized dictionaries were prepared for glycans, epitopes, and SNFG images of glycans, which were set up in PubDictionaries. While the glycan and epitope dictionaries facilitate semantic mapping to database IDs, the image dictionary provides a novel layer of visual metadata, mapping nomenclature to standardized SNFG representation. On the PubAnnotation side, the abstracts of all the articles from 15 selected high-impact journals in the field were prepared for annotation, and then they were comprehensively annotated using the dictionaries prepared in PubDictionaries. This workflow, executed via PubAnnotation, provides a dual-purpose enrichment of the literature:

- Database Interoperability: Term annotations serve as active hyperlinks to corresponding database entries, bridging the gap between literature text and structured data.
- Structural Clarity: SNFG image annotations provide cognitively enhanced recognition, allowing researchers to instantly grasp complex carbohydrate architectures that are often obscured by technical nomenclature.

The annotations were converted into RDF statements, enabling complex SPARQL queries across the dataset. This indexed repository is now integrated into GlyCosmos, with all annotations accessible for interactive visualization via TextAE.

The GlyCosmos Glycoscience Portal (Yamada et al. 2020; Lee et al. 2025) is a web portal for a plethora of data related to glycans. In particular, data from major life science resources including UniProt (UniProt 2025), Reactome (Alexander Grentner et al. 2024), KEGG GLYCAN (Kanehisa 2017), and other glycan-related resources are integrated. Glycans are retrieved from the GlyTouCan repository (Fujita et al. 2021) on a weekly basis, and each entry is annotated with additional metainformation such as monoisotopic mass, motifs, species information, etc.

While GlyCosmos attempts to accumulate as much comprehensive information about glycans as possible, it is not easy to extract the latest information from publications. Such extraction involves the selection of the right publication from pertinent journals, formatting the data in an appropriate manner, and incorporating the data into GlyCosmos in the relevant data resources.

In order to keep up with the latest publications, as a first step, we have incorporated PubAnnotation into GlyCosmos, extracting text from the latest literature containing pertinent keywords for diseases, organisms and anatomies related to glycans. Here, we describe the efforts we have undertaken to enable this process to be performed semi-automatically. We describe use-cases to illustrate its utility, and future work will discuss our plans for developing pipelines that can supplement the Data Resources in GlyCosmos based on these annotations.

## Results

### Dictionaries

The literature annotation resources for glycobiology were developed using a dual-strategy approach, prioritized by data availability and sustainability:

1. Integration of Established Resources: Existing high-quality annotation datasets were reused provided they were readily available and demonstrated a commitment to stable, long-term updates.
2. Agile Development of New Resources: In cases where high-quality datasets were unavailable or their maintenance status was uncertain, new resources were developed using the agile text mining method (Kim et al. 2019).

The agile text mining method is a dictionary-driven framework that bypasses the need for extensive gold-standard training data. By leveraging domain-specific dictionaries, this approach allows for the rapid generation and refinement of annotations, making it particularly effective for specialized fields like glycobiology where traditional training sets are scarce.

Initially, PubTator was considered as a source for reusable species and disease annotations which we needed. Ultimately, however, we chose to generate all the annotations using dictionary-based agile methodology for two primary reasons. First, while PubTator provides disease annotations, they are based on MeSH, whereas our system requires MONDO-based annotations to ensure interoperability with our other databases. Second, after a comparative performance evaluation of organism annotations between PubTator and the agile methodology, we concluded that the dictionary-based approach was better suited to our requirements. For a detailed performance analysis, please refer to the “Methodology” section.

Two custom dictionaries for glycan motifs and epitopes were prepared, and six more dictionaries were prepared for relevant biological entities based on the following sources:

- NCBITaxon (NCBI Taxonomy): A curated classification and nomenclature for all of the organisms in public sequence databases (Federhen 2012).
- MONDO (Mondo Disease Ontology): A semi-automatically constructed, community-driven ontology that merges multiple disease resources to harmonize and unify disease definitions across databases (Haendel et al. 2018).
- HPO (Human Phenotype Ontology): A structured and controlled vocabulary for the phenotypic abnormalities encountered in human disease (Köhler et al. 2014).
- FMA (Foundational Model of Anatomy): A reference ontology for human anatomy that symbolically represents the canonical spatial-structural entities and relationships forming the organization of the human body (Rosse and Mejino 2003)
- UBERON (Uber-anatomy ontology): An integrated, cross-species anatomy ontology offering a unified representation of anatomical structures across animals, organized by traditional classification, part-of, and developmental relationships (Mungall et al. 2012)
- MAT (Minimal Anatomical Terminology): A minimal core ontology providing a concise set of anatomical terms intended as a foundational vocabulary for anatomy [http://obofoundry.org/ontology/mat]

These eight dictionaries were compiled and loaded into PubDictionaries, as listed in Table 1. PubDictionaries is a repository of dictionaries, where a dictionary is a collection of mappings between natural language (NL) terms and identifiers. NL terms make interfaces more human-friendly but are prone to ambiguity (polysemy) and morphological variation (polymorphism). Identifiers, by contrast, provide unambiguous, machine-readable references but lack intuitiveness for human users. By maintaining mappings between NL terms and identifiers, PubDictionaries bridges the gap between human and machine interfaces, enabling seamless, precise communication across both domains. In PubDictionaries, multiple mappings with the same identifiers, but with different NL terms, represent polymorphism, and multiple mappings with the same NL term, but with different identifiers, represent polysemy. For the dictionaries loaded in it, PubDictionaries provides lookup services in both directions: search for identifiers by NL terms, and search for NL terms by identifiers. Also, PubDictionaries provides a text annotation service, which takes a block of text, finds NL terms in the text, and annotates them with the corresponding identifiers. For the lookup and annotation services, PubDictionaries can search for not only exact matches, but also morphologically similar terms. The Jaccard coefficient [Cohen, William W., Pradeep Ravikumar, and Stephen E. Fienberg. “A Comparison of String Distance Metrics for Name-Matching Tasks.” IIWeb. Vol. 3. 2003] is used for the similarity computation after standard syntactic normalization, including ICU tokenization [ICU User Guide – Boundary Analysis. Unicode Consortium, https://unicode-org.github.io/icu/userguide/boundaryanalysis.html] and Porter stemming [doi:10.1108/eb046814]. The text annotation component of PubDictionaries also features automatic abbreviation processing, which finds locally-defined abbreviations in a block of text, and maintains them as additional entries during the annotation of the text. While the official service of PubDictionaries is maintained at https://pubdictionaries.org, the source code is also freely available under MIT license (https://https://github.com/pubannotation/pubdictionaries).

**Table 1:** The dictionaries developed in this work. The type of entries stored in each dictionary, the number of entries, and the source of entries are listed.

**Dictionary**	**Entry type**	**# Entries**	**Source**	**Note**
Glycan	Glycan motifs	219	GlyCosmos Glycan DB	Glycan substructures identified as motifs in GlycoNAVI (last update Nov. 14, 2014)
GlycoEpitope	Glyco epitopes	723	GlyCosmos Epitope DB	Glycan epitopes as organized in GlyCosmos (last update Nov. 18, 2015)
NCBITaxon	Organisms	931,401	BioPortal	The entire tree of NCBI Taxonomy, version 2024 May 6.
MONDO	Diseases	112,359	BioPortal	The “Disease” subtree of the MONDO ontology, version 2025-01-07.
HPO	Phenotypes	39,555	BioPortal	The “Phenotypic abnormality” subtree of the HP ontology, version 2024 August 13.
FMA	Anatomy	147,341	BioPortal	The “Physical anatomical entity” subtree of the FMA ontology, version 5.0.0.
UBERON-AE	Anatomy	13,296	BioPortal	The “anatomical entity” subtree of UBERON ontology, version 2023 July 25.
MAT	Anatomy	558	BioPortal	The entire tree of the MAT ontology, the last version, archived in OBO Foundry.

### Literature collection and annotations

Regarding the source of literature collection for glycobiology, we chose the following 15 journals:

- Analytical Chemistry
- Biochimica et Biophysica Acta
- Carbohydrate Research
- Cell
- Glycobiology
- Glycoconjugate Journal
- Journal of the American Chemical Society
- Journal of Biological Chemistry
- Journal of Proteome Research
- Journal of Proteomics
- Molecular and Cellular Proteomics
- Nature Biotechnology
- Nature Communications
- Nature Methods
- Scientific Reports

All abstracts from these journals were collected from PubMed, which resulted in 650,028 documents. As of the time of writing, this collection covers publications up to 2025 April 20. Using the Glycan and GlycoEpitope dictionaries, we annotated glycan motifs and epitopes, yielding 43,335 glycan annotations and 28,481 epitope annotations across 15,463 documents. We then annotated the same set of documents using the other six dictionaries. In total, this resulted in annotations for eight types of biological entities. Finally, to facilitate human-friendly, sentence-level access to the annotated text, we performed sentence segmentation, producing 141,265 segmented sentences. The annotation results are summarized in Table 2.

**Table 2:** The result of annotations using the dictionaries in Table 1. For each entity type, the number of annotations detected across all of the selected journals and the top five most frequent terms are listed.

**Entity type (Dictionary)**	**# Annotations**	**Five most frequently annotated terms**
Glycan motifs (Glycan)	45,099	GalNAc (4175), Chondroitin (3999), Neu5Ac (2535), AGM3 (2292), chitin (2018)
Glyco epitopes (GlycoEpitope)	28,481	Chondroitin (4001), GM1 (1943), O-GlcNAc (1749), Heparan Sulfate (1403), T Antigen (895)
Taxon (NCBITaxon)	37,463	*Homo sapiens* (2532), Mus (1400), Bacillus coli (806), Mouse (700), Rattus (696)
Diseases (MONDO)	20,306	Neoplasm (1912), Cancer (1507), Infectious disease (898), Cholera (443), Melanoma (441)
Phenotypes (HP)	13,792	Neoplasm (2363), Carcinoma (359), Melanoma (316), Breast cancer (284), Alzheimer disease (248)
Anatomy (FMA)	54,506	Portion of tissue (2020), Brain (1228), Cell surface (1152), Blood (1138), Blood serum (938)
Anatomy (UBERON)	58,499	Membrane (2617), Brain (1213), Blood (1099), Tissue (1050), Liver (895)
Anatomy (MAT)	21,222	Brain (1338), Blood (1160), Liver (948), Cartilage (846), Gut (741)

Note that these statistics are based on the ontology identifiers, not the terms themselves. Thus, for example, the 1215 annotation instances of “Neoplasm” (MONDO:0005070) based on the MONDO dictionary included annotations to its synonyms registered in the dictionary, which are as follows:

- neoplasm
- cell process disease
- tumor disease
- tumor
- tumour disease
- neoplastic growth
- tumour
- neoplastic disease
- disease of cellular proliferation
- neoplasia

As disease and phenotype are close concepts to each other, we performed a comparative analysis on the annotation results. The following is a deeper comparison of the annotations with a focus on the five most frequent annotated entities based on the MONDO and HP dictionaries.

- While MONDO distinguishes neoplasm (MONDO:0005070) and cancer (MONDO:0004992), HP treats them as the same term (HP:0002664).
- In the case of “Carcinoma” (HP:0002664), HP-based annotation produced substantially more instances than MONDO-based annotation. Further analysis showed that MONDO subdivides Carcinoma into finer-grained terms — such as colon carcinoma (MONDO:0002032) — thereby dispersing annotations across these subconcepts.
- In the case of infectious disease, both MONDO and HP include fine-grained terms for specific infectious diseases, but only MONDO defines the umbrella term “infectious disease.”
- HPO does not include a term for “Cholera”; instead, it imports this concept from ORDO. Therefore, when annotating with HP, supplementing it with ORDO may ensure better coverage of diseases.

These annotation results were next converted into RDF statements and stored in a SPARQL endpoint (https://ep3.pubannotation.org/sparql), which enables richer search through SPARQL queries. The RDF representation of the annotations is illustrated with an example in Figure S1. For users who are not familiar with SPARQL, query templates are also available with fillable blanks for specific query terms (https://pubannotation.org/collections/GlyCosmos15/search). For example, in the following, the underlined terms can be replaced by a relevant term to search for:

1. Retrieve the statistics of all the annotated terms
2. Retrieve the statistics of all the annotated terms in the dictionary __DICTIONARY__ that co-occur with glycans in the same sentences.
3. Find all the sentences with annotations for __URL__, and show them in TextAE
4. Find all the sentences with annotations for __URL1__ and __URL2__, and show them in TextAE
5. Find all the sentences with both Glycan and disease (MONDO) annotations, and show them in TextAE

**Figure 1:**
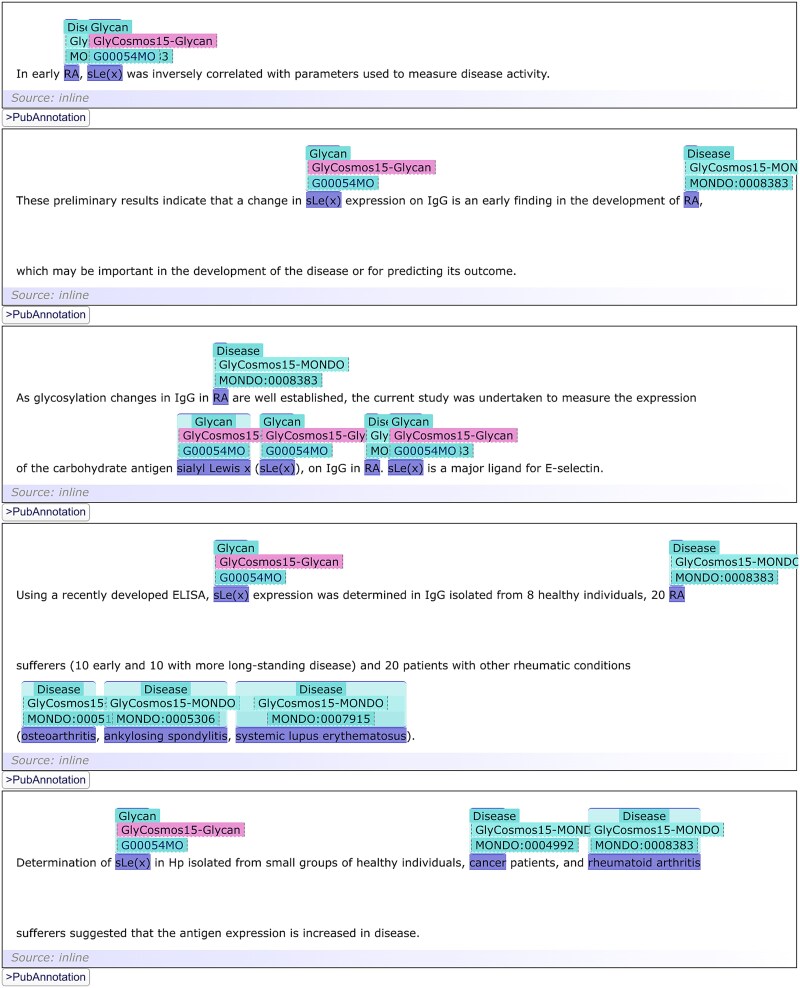
Visualization of the search results for query template 4. The results demonstrate the co-occurrence of glycan:G00054MO (sialyl-Lewis a) and MONDO:0008383 (rheumatoid arthritis), rendered via TextAE. For clarity, the view is filtered to display only the glycan and MONDO annotation layers.

The first two can be used to obtain statistics on annotated terms in varying conditions. The last three can be used to retrieve actual annotations rendered in TextAE: the third one for a specific term identified by the URL, the fourth one for two co-occurring specific terms identified by URL1 and URL2, and the fifth for co-occurrences of a glycan and a disease. Figure 1 shows the results of using query template 4, with URL1 and URL2 set to Glycan:G00054MO (sialyl Lewis X) and MONDO:0008383 (rheumatoid arthritis), respectively, demonstrating potential useful usages of the annotated resources. This result was produced from the query displayed in Figure S2. Note that, to conserve space, throughout this manuscript, prefixes are used in place of full URLs, for example, “Glycan” for ‘https://glycosmos.org/glycans/show/’ and “MONDO” for ‘http://purl.obolibrary.org/obo/MONDO_’. For reproducibility, all annotations are accessible via the “GlyCosmos15” collection on PubAnnotation (https://pubannotation.org/collections/GlyCosmos15). This collection comprises eight projects, each corresponding to a specific type of annotation, along with one project that includes all documents without annotations and another for sentence segmentation annotations. Each project specifies the exact API used to obtain its annotations, including the parameters applied.

### Integration into the GlyCosmos portal

**Figure 2:**
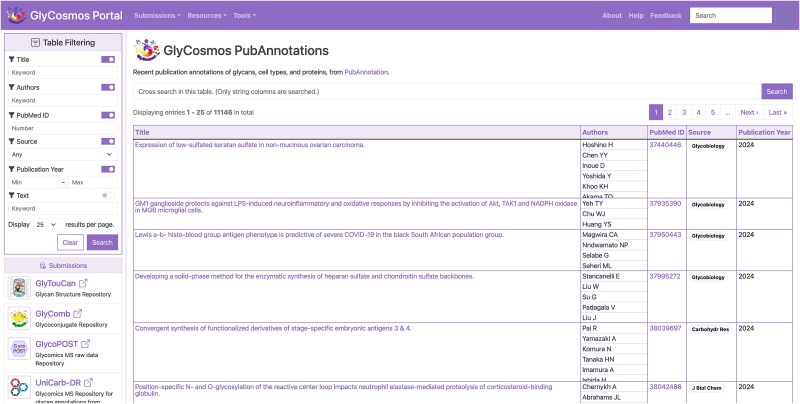
List of articles in GlyCosmos PubAnnotation.

**Figure 3:**
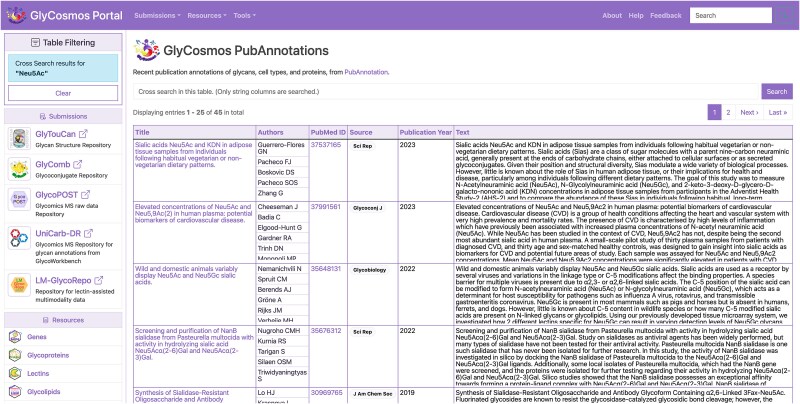
Results of keyword search for “Neu5Ac” in GlyCosmos PubAnnotation.

**Figure 4:**
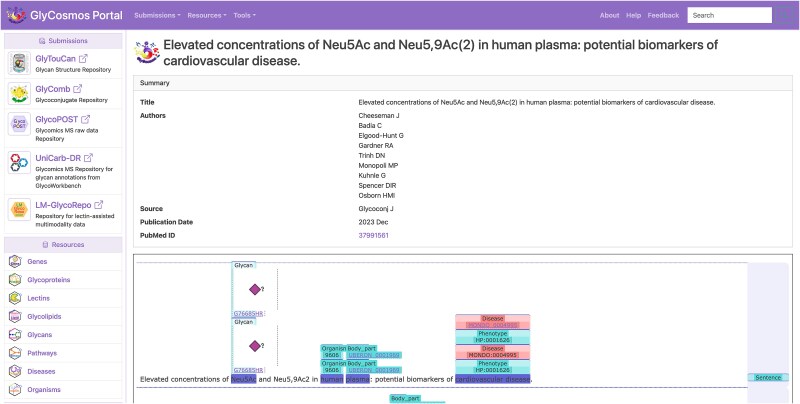
Detail page of an article (PMID:37991561) in GlyCosmos PubAnnotation, displayed using TextAE.

The list of articles with annotations related to glycobiology obtained as a result of searching the latest literature at the time of the last update can be found at GlyCosmos PubAnnotation (https://glycosmos.org/pubannotations). This page contains a table of the articles retrieved from PubMed, including their titles, authors’ names, publication year, etc (Fig. 2). The table supports, for example, keyword searches in each column and filtering by year of publication. Figure 3 shows some of the results of a search of PubMed abstracts with the keyword “Neu5Ac.” Then, clicking on any article title link in this table will open a detail page displaying all the annotations for the article, rendered in TextAE (Fig. 4).

**Figure 5:**
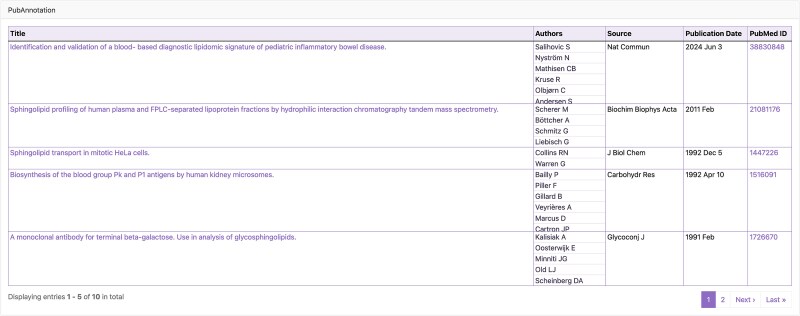
List of articles associated with “G84224TW” in GlyCosmos glycans. This was made possible by the use of semantic web technologies.

In addition, each GlyCosmos Glycan entry page presents a list of articles in which that specific glycan appears as an annotation. These articles link back to GlyCosmos PubAnnotation, offering seamless navigation between glycan data and the related literature (Fig. 5).

## Discussion

In this work, we presented the results of a collaboration between GlyCosmos and PubAnnotation, whereby all the abstracts from selected journals were compiled as relevant literature resources of glycobiology, and they were annotated for glycan motifs and epitopes, together with other biologically relevant entities like taxonomy, diseases and anatomical structures. The resulting annotations were stored as RDF statements such that they can be accessed across the Semantic Web and queried through SPARQL. A user-friendly Web interface in GlyCosmos is also provided for biologists to easily view the results and links to their corresponding pages in GlyCosmos Glycans for glycans, GlyCosmos Organisms for taxonomies, OBO for UBERON anatomies, and MONDO for diseases.

We showed an example of how specific paragraphs or sentences from relevant literature can be obtained and viewed, highlighted with glycan images in SNFG, and linked to one another. PubAnnotation also provides useful features such as recognizing abbreviations for glycan epitopes, such as “sLex” for “sialyl Lewis X” or even abbreviations specified by authors earlier in the text, such as in the example of Rheumatoid Arthritis (RA), with “RA” automatically being highlighted independently, later in the text. Another feature is the specification of term “thresholds” whereby variations of a term can automatically be detected. For example, “sialylated Lewis A” is not registered in our dictionary, but it could be annotated because “sialyl Lewis A” is defined, and the threshold of .91 allows it to be found. Thus, the preparation of dictionaries has become much easier as it does not require users to list all possible variations of a term in the dictionary.

We also showed that various search results suggest insight into glycan motifs and other biological entities. For example, based on our resulting annotations for the Human Phenotype Ontology (HP), it is apparent that glycan research is often related to cancer and Alzheimer’s disease. However, the MONDO ontology also includes terms for infection and cholera. On the other hand, both have an overwhelming number of annotations for “neoplasm” indicating the importance of glycans in neoplasm. For glycans, Chondroitin was also a highly-frequent term.

This result shows the effect of using different ontologies for the same type of entities, e.g. MONDO and HP ontologies for disease terms.

The results of the search for co-occurrences of specific glycan motifs and diseases (Table 3; produced by query in Figure S3) suggest literature-supported associations of these two types of entities. In this table, GlyTouCan IDs are provided as a reference to the specific glycan structure. The most frequent pair is “cholera” and “GM1” which is well known as cholera toxin binds specifically to the GM1 ganglioside. The same can be said for influenza and sialic acid (Neu5Ac). The specific publications where these pairs occur frequently can be investigated in more detail from GlyCosmos.

**Table 3:** The top 15 most frequently co-occurring pairs of glycan motifs and diseases. GlyTouCan IDs are provided as a reference to the specific glycan structure. The most frequent pair is “cholera” and “GM1” which is well known as cholera toxin binds specifically to the GM1 ganglioside. The same can be said for influenza and sialic acid (Neu5Ac). The specific publications where these pairs occur frequently can be seen from GlyCosmos.

**Glycan motif**	**Disease**	**Count**
GM1 (Glycan:G48558GR)	cholera (MONDO:0015766)	162
GalNAc (Glycan:G39738WL)	cancer (MONDO:0004992)	69
Chondroitin (Glycan:G43702JT)	Ehlers-Danlos syndrome, spondylodysplastic type (MONDO:0007526)	56
Chondroitin (Glycan:G43702JT)	dermatan sulphate proteoglycan (MONDO:0020682)	55
GD3 (Glycan:G98544DH)	melanoma (MONDO:0005105)	52
Neu5Ac (Glycan:G76685HR)	glutaryl-CoA dehydrogenase deficiency (MONDO:0009281)	52
GM3 (Glycan:G91237TK)	cancer (MONDO:0004992)	45
GalNAc (Glycan:G39738WL)	neoplasm (MONDO:0005070)	42
T Antigen (Glycan:G00031MO)	neoplasm (MONDO:0005070)	37
GD2 (Glycan:G02657AK)	neuroblastoma (MONDO:0005072)	37
Sialyl Lewis x (Glycan:G00054MO)	cancer (MONDO:0004992)	37
GM3 (Glycan:G91237TK)	neoplasm (MONDO:0005070)	36
Chondroitin (Glycan:G43702JT)	melanoma (MONDO:0005105)	34
Neu5Ac (Glycan:G76685HR)	influenza (MONDO:0005812)	33
Chondroitin (Glycan:G43702JT)	neoplasm (MONDO:0005070)	33

From the database developer’s perspective, one can see how such analytical results can open up the possibility of enriching glycan databases with disease association information. Note that the association between two different types of entities, as shown in Table 3, can be obtained between any pair of the eight types of entities annotated in the literature resources. This multi-dimensional association space opens up the possibility of applying link discovery methods – such as matrix factorization – to uncover latent relationships, an avenue we reserve for future work.

### Regular updates of the literature annotations

GlyCosmos data are updated every four months; thus the process of updating the annotations of the latest literature using PubAnnotation also runs on a four-month cycle. Upon every update, the dictionaries used in PubDictionaries can also be manually updated with the latest terms as needed. User feedback is also welcome; any new dictionaries that would be of use for the community can be easily incorporated into PubAnnotation and made available in GlyCosmos.

Conversely, the Data Resources can be supplemented based on the results from PubAnnotation by adding links to the relevant paragraphs in PubAnnotation from each data entry, such as each GlyTouCan ID in GlyCosmos Glycans and each taxonomy entry page in GlyCosmos Organisms. Since these annotations are stored in RDF, the next update of GlyCosmos can include such references from these pages. Furthermore, terms that co-occur often in the literature can also be retrieved from the RDF data. For example, glycans and diseases, or glycans and terms of anatomies that frequently co-occur in the same text can be searched.

### Significance of this work

One of the largest bottlenecks for database developers (and consequently AI researchers) is the curation of the literature in order to populate databases with accurate and referenceable information. The work presented in this manuscript allows researchers to easily obtain annotations from the latest publications and view them in a user-friendly interface (via TextAE). Compared to other publication annotation tools such as PubTator, the agile approach supported by PubAnnotation offers two key advantages. First, it enables rapid addition of new annotations through simple dictionary engineering, without the need for machine learning. This is especially useful when machine learning resources, such as training data, are not readily available. Second, PubAnnotation provides infrastructure for a smooth transition from dictionary-based annotation to storing the results as RDF statements, facilitating integration with the Semantic Web and the Linked Open Data resources. Since GlyCosmos also uses RDF, this makes it easy to incorporate the annotation results into the respective entry pages for glycans and diseases, etc.

While the current dictionaries are limited to glycans, anatomies and diseases, with the simple addition of dictionaries by which to search, other related fields such as microbes, viruses, plant biology, materials, etc. can be annotated in a straightforward manner with this system. GlyCosmos is always open to new suggestions. As such, a feedback form is available for any interested users to suggest new datasets and fields of research to incorporate into its resources.

### Limitations

The current annotation process does not fully account for entities appearing in elliptical coordination or as coreference, both of which are recognized as significant challenges in the field of named entity recognition (NER). Because the primary goal of this study was to develop literature resources using the current state-of-the-art, we adopted a pragmatic strategy: we will import readily available annotations where possible or utilize a dictionary-based agile approach — specifically PubDictionaries — when dedicated training resources are unavailable.

While PubDictionaries and PubTator are highly effective for standard entity recognition, their performance is limited by the discontinuity inherent in elliptical structures. In these cases, essential parts of an entity name are omitted (e.g. in “HPV-16 or -18,” the species reference “-18” is a fragment that lacks the preceding genus and species identifiers), leading to information loss because standard systems are generally designed to identify complete, continuous text spans. Pushing performance beyond the current state-of-the-art to effectively resolve these elided and coreferential entities remains a key objective for future work.

## Materials and methods

### Agile development of dictionaries and annotations

The dictionaries and the literature annotation resources for GlyCosmos were developed following the agile annotation method proposed in (Kim et al. 2019). First, the dictionaries for glycan motifs (Glycan) and GlycoEpitopes were developed by sourcing the terms from the GlycoNAVI (https://glyconavi.org) and GlycoEpitope databases (https://www.glycoepitope.jp/) respectively. The other dictionaries were developed based on corresponding ontologies in BioPortal, a repository of biomedical ontologies. This approach simplifies accessing various sources of dictionaries, as we can access all the ontologies through the same application programming interface (API). To ensure domain specificity, targeted retrieval of terms from each ontology was performed by subsetting only relevant branches. For FMA as an example, terms categorized under “Physical anatomical entity” were extracted while the “Biological macromolecule” subtree was explicitly extracted to maintain a focus on anatomical structures. Synonyms were harvested by collecting the values from the “prefLabel” and “altLabel/synonym” properties. To ensure reproducibility, these selection criteria were documented within the metadata for each individual dictionary in PubDictionaries.

Annotations were made using the text annotation functionality of PubDictionaries, which is based on string matching and string similarity computation. The initial versions of dictionaries, for which the entries were collected from databases and ontologies, were error-prone, and we conducted a filtering process following the agile development methodology. Following this method, dictionaries were refined through multiple iterations; 10 randomly selected documents were annotated by the dictionary, false positives (FPs) and false negatives (FNs) were identified, error-prone entries were removed, and new synonyms were added to amend the false positives and false negatives. The threshold of string similarity was also adjusted. These iterations continued until no more false positives or false negatives were found. In practice, all the dictionaries reached stability within fewer than 10 iterations. Nevertheless, the interaction was continued to 10 iterations to ensure additional validation.

Even after this initial stabilization, the dictionaries remained dynamic. As further refinements were identified during broader application, ad-hoc additions or deletions were integrated to improve subsequent annotation cycles. A key feature of the PubDictionaries architecture is its ability to maintain a comprehensive registry of these modifications, specifically as “white entries” (custom additions) and “black entries” (custom deletions). This mechanism ensures that when dictionaries are synchronized with newer versions of their source ontologies, these manual refinements are preserved and seamlessly re-applied, maintaining the consistency and integrity of the annotations over time.

**Figure 6:**
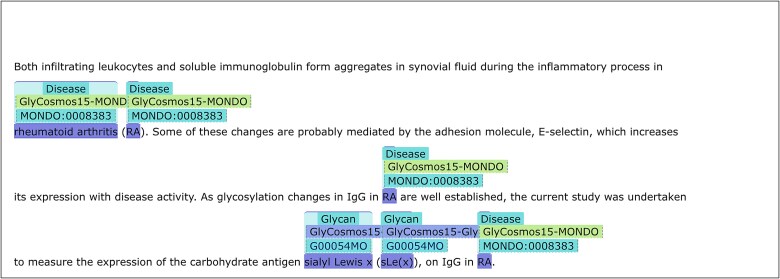
Annotation with locally defined abbreviations. The abbreviations “RA” and “sLe(x)” are not in the MONDO and glycan dictionaries, but they are recognized as abbreviations for “rheumatoid arthritis” and “sialyl Lewis x”, respectively, and as such they are annotated properly.

When terms including synonyms are collected from databases or ontologies, the most error-prone entries are short abbreviations. Thus, abbreviations shorter than 4 letters were all removed from the dictionaries. Instead, PubDictionaries can recognize locally defined abbreviations. Figure 6 is an example.

**Figure 8:**
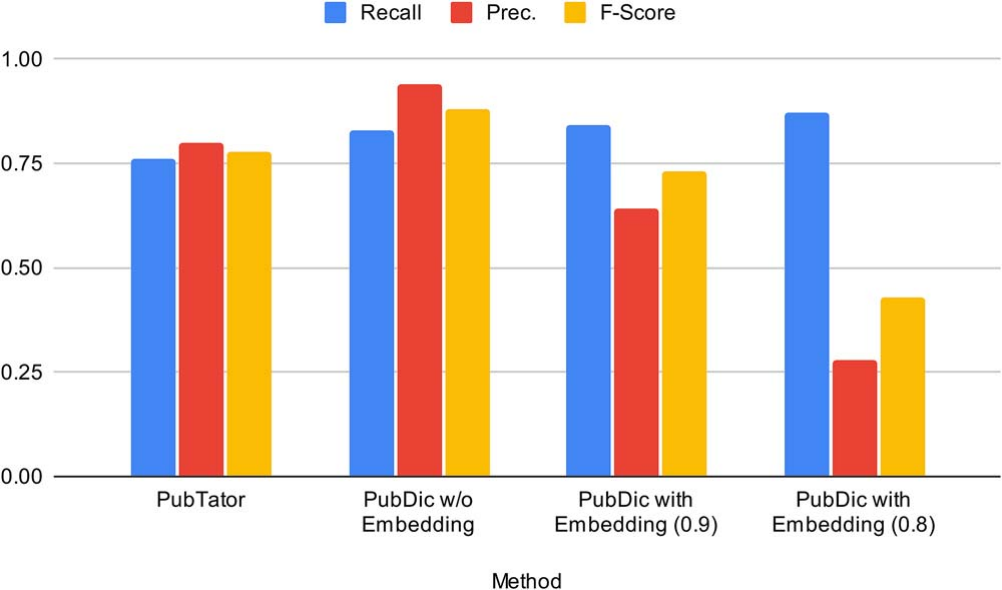
Performance comparison of PubTator and PubDictionaries with and without use of embedding models.

**Figure 7:**
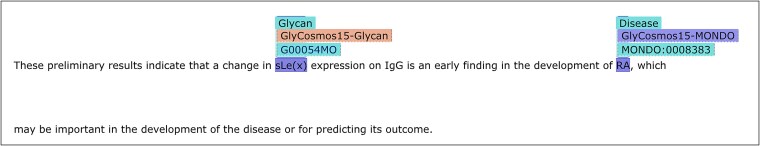
Annotation with locally defined abbreviations. Abbreviations such as “sLe(x)” and “RA” could be annotated because their definitions appeared elsewhere within the same document.

In Fig. 6, while “RA” is not in the “MONDO” dictionary, PubDictionaries recognizes the local definition of the abbreviation in the pattern “rheumatoid arthritis (RA)” and annotates all the appearances of “RA” using the same identifier as “rheumatoid arthritis” (MONDO:0008383). The same applies to the local definition of the abbreviation “sLe(x)” in “sialyl Lewis x (sLe(x))”. This function is useful to increase the sensitivity of annotation, as it is often that after abbreviations are defined, only the abbreviations are used later in the same document, as exemplified in Fig. 7.

The annotation quality of the dictionary-based agile methodology was assessed through organism-specific annotation. To establish a benchmark, 50 documents were randomly sampled and annotated using two sources: PubTator and PubDictionaries. These annotations were manually reviewed and reconciled to create a reference set. It should be noted that some annotations may be missing if they were overlooked by both systems; consequently, the results are intended primarily for a comparative evaluation of the two methods. PubDictionaries was evaluated across multiple configurations to compare its default setting (no embeddings) against its embedding-based semantic capabilities for automated synonym expansion.

Table 4 and Fig. 8 shows the result of the evaluation. As shown in the first and second rows, the default configuration of PubDictionaries outperformed PubTator in both precision and recall. A detailed analysis provided the following insights:

- PubTator’s lower recall is largely attributed to its convention not to annotate nested entities. For instance, “human” in “Type II human complement C2 deficiency” is not annotated because it is embedded within the disease name.
- PubTator occasionally annotates terms without normalizing them to unique identifiers. While it can identify novel entities not present in the dictionary, these are often highly erroneous, leading to lower precision.

**Table 4:** Performance comparison of PubTator and PubDictionaries. Comparison against gold-standard annotations for 50 randomly sampled documents. PubDictionaries performance is evaluated under three configurations: no embedding (system default), and embedding-based semantic similarity with thresholds of 0.9 and 0.8.

Method	TPs	FNs	FPs	Recall	Prec.	F-Score
PubTator	88	28	22	0.76	0.80	0.78
PubDic w/o Embedding	96	20	6	0.83	0.94	0.88
PubDic with Embedding (0.9)	97	19	54	0.84	0.64	0.73
PubDic with Embedding (0.8)	101	15	254	0.87	0.28	0.43

Based on this comparison, we elected to generate annotations using PubDictionaries rather than re-using PubTator data. Furthermore, as shown in the final two rows of Table 4, while the use of embeddings slightly improved recall, it introduced significant noise (false positives). These results suggest that while semantic similarity is a valuable tool for synonym discovery, it is currently unsuitable for automated, production-grade annotation. To ensure transparency and reproducibility, all datasets and results, including the lists of true positives, false positives, and false negatives, of the comparative evaluation are publicly available via PubAnnotation (https://pubannotation.org/collections/Organism-Comparison).

## Supplementary Material

PubAnnotation_in_Gly_Cosmos_paper_Supplementary_Material_cwag015

## Data Availability

The data presented as a result of this work is available via the GlyCosmos Portal under a CC-BY-4.0 license. The dictionaries and annotations available via PubAnnotation and PubDictionaries are also under a CC-BY-4.0 license.

## References

[ref2] Federhen S . The NCBI taxonomy database. Nucleic Acids Res. 2012:40(D1):D136–D143. 10.1093/nar/gkr1178. 22139910 PMC3245000

[ref3] Fujita A, Aoki NP, Shinmachi D, Matsubara M, Tsuchiya S, Shiota M, Ono T, Yamada I, Aoki-Kinoshita KF. The international glycan repository GlyTouCan version 3.0. Nucleic Acids Res. 2021:49 (D1):D1529–D1533. 10.1093/nar/gkaa947. 33125071 PMC7779025

[ref4] Grentner A, Ragueneau E, Gong C, Prinz A, Gansberger S, Oyarzun I, Hermjakob H, Griss J. ReactomeGSA: new features to simplify public data reuse. Bioinformatics. 2024:40(6):btae338. 10.1093/bioinformatics/btae338. PMC11147800 38806182

[ref5] Haendel MA, McMurry JA, Relevo R, Mungall CJ, Robinson PN, Chute CG. A census of disease ontologies. Annu Rev Biomed Data Sci. 2018:1(1):305–331. 10.1146/annurev-biodatasci-080917-013459.

[ref6] Kanehisa M . KEGG glycan. In: Aoki-Kinoshita KF, editors. A practical guide to using Glycomics databases. Tokyo: Springer Japan; 2017. pp. 177–193. [accessed 2025 Jun 3] 10.1007/978-4-431-56454-6_9

[ref7] Kim J-D, Wang Y, Fujiwara T, Okuda S, Callahan TJ, Cohen KB. Open agile text mining for bioinformatics: the PubAnnotation ecosystem. Bioinformatics. 2019:35(21):4372–4380. 10.1093/bioinformatics/btz227. 30937439 PMC6821251

[ref8] Köhler S, Doelken SC, Mungall CJ, Bauer S, Firth HV, Bailleul-Forestier I, Black GCM, Brown DL, Brudno M, Campbell J, et al. The human phenotype ontology project: linking molecular biology and disease through phenotype data. Nucleic Acids Res. 2014:42(D1):D966–D974. 10.1093/nar/gkt1026. 24217912 PMC3965098

[ref9] Lee S, Ono T, Masaaki S, Fujita A, Matsubara M, Zappa A, Yamada I, Aoki-Kinoshita KF. Updates implemented in version 4 of the GlyCosmos Glycoscience portal. Anal Bioanal Chem. 2025:417(5):907–919. 10.1007/s00216-024-05692-0. 39690313 PMC11782317

[ref10] Mungall CJ, Torniai C, Gkoutos GV, Lewis SE, Haendel MA. Uberon, an integrative multi-species anatomy ontology. Genome Biol. 2012:13(1):R5. 10.1186/gb-2012-13-1-r5. 22293552 PMC3334586

[ref11] Rosse C, Mejino JLV. A reference ontology for biomedical informatics: the foundational model of anatomy. J Biomed Inform. 2003:36(6):478–500. 10.1016/j.jbi.2003.11.007. 14759820

[ref1] UniProt . The universal protein knowledgebase in 2025. Nucleic Acids Res. 2025:53(D1):D609–D617. 10.1093/nar/gkae1010. 39552041 PMC11701636

[ref12] Yamada I, Shiota M, Shinmachi D, Ono T, Tsuchiya S, Hosoda M, Fujita A, Aoki NP, Watanabe Y, Fujita N, et al. The GlyCosmos portal: a unified and comprehensive web resource for the glycosciences. Nat Methods. 2020:17(July):649–650. 10.1038/s41592-020-0879-8. 32572234

