# Supplementary material for: Resources for screening the literature for glycan-related terms using PubAnnotation in GlyCosmos
Source: Glycobiology. 2026 Mar 9;36(5):cwag015. doi: 10.1093/glycob/cwag015 (PMC13011802; doi:10.1093/glycob/cwag015)
Supplement: PubAnnotation_in_Gly_Cosmos_paper_Supplementary_Material_cwag015 [file pubannotation_in_gly_cosmos_paper_supplementary_material_cwag015.docx]

## Supplementary Material

Resources for screening the literature for glycan-related terms using PubAnnotation in GlyCosmos

Running head: PubAnnotation in GlyCosmos

# Author list

Jin-Dong Kim^1^, Masaaki Shiota^2^, Issaku Yamada^3^, Kiyoko F. Aoki-Kinoshita^2,*^

Affiliations:

1. Database Center for Life Science (DBCLS), Research Organization of Information and Systems (ROIS), 178-4-4 Wakashiba, Kashiwa, Chiba 277-0871, Japan
2. Glycan and Life Systems Integration Center (GaLSIC), Soka University, 1-236 Tangi-machi, Hachioji, Tokyo 192-8577, Japan
3. The Noguchi Institute, 1-9-7 Kaga, Itabashi, Tokyo 173-0003, Japan

- Corresponding author: 1-236 Tangi-machi, Hachioji, Tokyo, Japan 192-8577, [kkiyoko@soka.ac.jp](mailto:kkiyoko@soka.ac.jp)

##

## RDF indexing and searching

PubAnnotation has a function to convert all of its annotations to RDF statements, which can then be loaded into a SPARQL endpoint (Virtuoso). Figure 8 shows example RDF statements describing a sentence which includes annotations for MONDO:0008383 (rheumatoid arthritis) and Glycan:G00054MO (Sialyl Lewis x). It represents the first result shown in Figure S-1. Figure S-2 shows a SPARQL query for searching all the sentences which have both the glycan sialyl Lewis x (Glycan:G0005MO) and the disease rheumatoid arthritis (MONDO:0008383). It produces the results shown in Figure 1. Figure S-3 shows a SPARQL query to get the statistics of the occurrence of glycans and diseases in the same sentences. It produces the results shown in Table 3. As discussed in Result, for the users who are not familiar with RDF, we also provide query templates. See the Result section for more details.

Figure S-1. Example of RDF representation in PubAnnotation
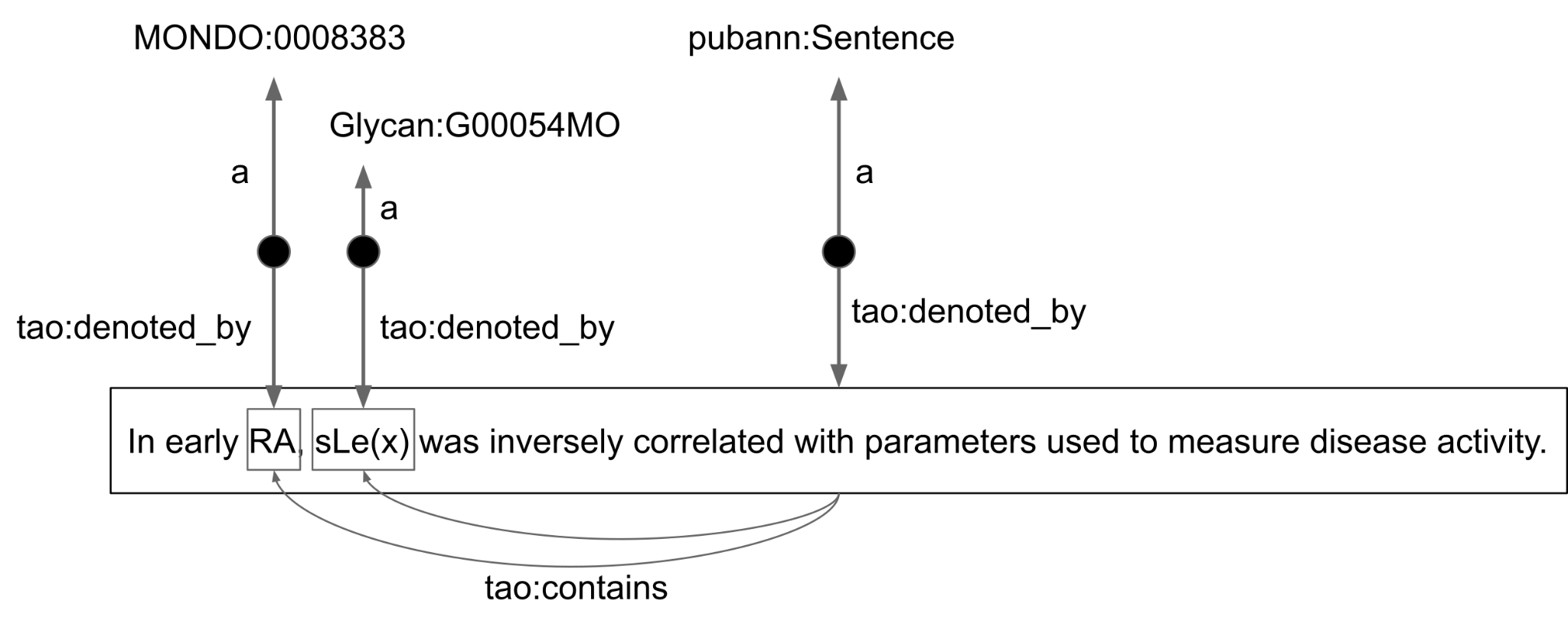


| PREFIX glycan: <https://glycosmos.org/glycans/show/>  PREFIX mondo: <http://purl.obolibrary.org/obo/MONDO_>  SELECT DISTINCT ?span3  WHERE {  ?object1 tao:denoted_by ?span1 ; a glycan:G00054MO .  ?object2 tao:denoted_by ?span2 ; a mondo:0008383 .  ?object3 tao:denoted_by ?span3 ; a pubann:Sentence .  ?span3 tao:contains ?span1 .  ?span3 tao:contains ?span2 .  } |
| --- |

Figure S-2. SPARQL query to produce the results in Figure 1

| SELECT ?id1 ?id2 (COUNT(*) AS ?count)  WHERE {  GRAPH prj:GlyCosmos15-Glycan {  ?o1 tao:denoted_by ?s1 ; a ?id1 .  }  GRAPH prj:GlyCosmos15-MONDO {  ?o2 tao:denoted_by ?s2 ; a ?id2 .  }  GRAPH prj:GlyCosmos15-Sentences {  ?o3 tao:denoted_by ?s3 .  }  ?s3 tao:contains ?s1 .  ?s3 tao:contains ?s2 .  FILTER (!CONTAINS(STR(?id1), "projects"))  FILTER (!CONTAINS(STR(?id2), "projects"))  }  GROUP BY ?id1 ?id2  ORDER BY DESC(?count) |
| --- |

Figure S-3. SPARQL query to produce the results in Table 3
